# Localization of polyhydroxybutyrate in sugarcane using Fourier-transform infrared microspectroscopy and multivariate imaging

**DOI:** 10.1186/s13068-015-0279-y

**Published:** 2015-07-10

**Authors:** Jason S. Lupoi, Andreia Smith-Moritz, Seema Singh, Richard McQualter, Henrik V. Scheller, Blake A. Simmons, Robert J. Henry

**Affiliations:** Queensland Alliance for Agriculture and Food Innovation, University of Queensland, St. Lucia, Queensland 4072 Australia; Joint BioEnergy Institute, Lawrence Berkeley National Laboratory, 5885 Hollis Street, Emeryville, CA 94608 USA; Biological and Engineering Sciences Center, Sandia National Laboratories, 7011 East Avenue, Livermore, CA 94551 USA; Australian Institute for Bioengineering and Nanotechnology, University of Queensland, St. Lucia, Queensland 4072 Australia; Department of Biological Sciences, University of North Texas, 1155 Union Circle #305220, Denton, TX 76203 USA; Sage Analytics, Boulder, CO 80301 USA

**Keywords:** Infrared imaging, Focal plane array, Polyhydroxybutyrate, Sugarcane, Multivariate imaging

## Abstract

**Background:**

Slow-degrading, fossil fuel-derived plastics can have deleterious effects on the environment, especially marine ecosystems. The production of bio-based, biodegradable plastics from or in plants can assist in supplanting those manufactured using fossil fuels. Polyhydroxybutyrate (PHB) is one such biodegradable polyester that has been evaluated as a possible candidate for relinquishing the use of environmentally harmful plastics.

**Results:**

PHB, possessing similar properties to polyesters produced from non-renewable sources, has been previously engineered in sugarcane, thereby creating a high-value co-product in addition to the high biomass yield. This manuscript illustrates the coupling of a Fourier-transform infrared microspectrometer, equipped with a focal plane array (FPA) detector, with multivariate imaging to successfully identify and localize PHB aggregates. Principal component analysis imaging facilitated the mining of the abundant quantity of spectral data acquired using the FPA for distinct PHB vibrational modes. PHB was measured in the chloroplasts of mesophyll and bundle sheath cells, acquiescent with previously evaluated plant samples.

**Conclusion:**

This study demonstrates the power of IR microspectroscopy to rapidly image plant sections to provide a snapshot of the chemical composition of the cell. While PHB was localized in sugarcane, this method is readily transferable to other value-added co-products in different plants.

## Background

The use of plastics has become ubiquitous in almost every facet of our lives due to low cost and versatile properties. The majority of plastics are obtained from fossil fuels, and as the price of crude oil continues to fluctuate, the price of plastics that are produced from oil feedstocks are expected to oscillate as well (http://articles.ides.com/oil.asp). Fossil fuel-derived plastics also degrade slowly in the environment producing deleterious effects, most notably in marine environments [1–3]. The need to find price-stable sources of plastic resin and to overcome the environmental problems is driving the search for bio-renewable, biodegradable plastics.

Polyhydroxybutyrate (PHB) is a biodegradable polyester which possesses similar physical properties to many petroleum-derived plastics [4]. PHB is generated naturally by micro-organisms as a form of energy storage during stress [5]. The biosynthetic pathway for PHB production has been isolated and used for the large-scale manufacture of PHB by fermentation. However, the high production cost of PHB produced by fermentation and the price of starting substrate pose significant drawbacks [6]. An alternative is to engineer PHB production in plants, which as autotrophs provide their own substrate and energy for PHB production in the form of carbon dioxide, water, and sunlight. The manufacturing of PHB as a value-added co-product in plants, particularly high-biomass-yielding crops such as sugarcane, switchgrass, and maize, has the potential to improve the economics of their use for bio-plastic fabrication [7, 8]. PHB has been successfully engineered in a number of plant species [9] and has potential applications not only as a bio-plastic but also for the manufacture of chemicals and improved animal feed [9–13].

The best results thus far in high-biomass grasses have been obtained by engineering PHB synthesis to the chloroplast [9, 14–16]. PHB production in the chloroplasts of C_4_ grasses resulted in polymer formation in all types of leaf cells, as identified by microscopic visualization of granules, with the exception of mesophyll cells where no or only few PHB granules were observed [16]. Strategies to increase polymer production in high-biomass grasses have included the use of stronger constitutive promoters [15], simultaneous overexpression of photosynthesis-related genes [9], as well as modification of the PHB biosynthetic pathway [16]. Although all of these strategies have increased polymer content, only the latter managed to utilize the full capacity of mesophyll cells. Because the spatial distribution of PHB within the sugarcane leaf is crucial to achieving high yields, a robust tool for quantifiably mapping the localization of PHB within the leaf would be of great value.

**Fig. 1:**
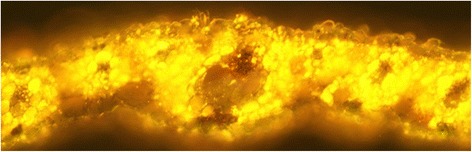
Example of the fluorescence generated when Nile Blue A staining is used to visualize lipids inside sugarcane

The physical presence of PHB granules in bacteria [17] and plants [18] has traditionally been detected by Nile Blue A staining. Nile Blue A staining is used in histology to highlight the distinction between neutral lipids which stain pink and acids which stain blue. When a 450-nm excitation wavelength was applied, the PHB granules stained with Nile Blue A showed a strong orange fluorescence (Fig. 1). While the technique is useful for screening for the presence of PHB granules in plant cells, it often produces false positives when polymer levels are low, due to its non-specificity [15]. To the untrained eye, waxy deposits on the leaf surface can often be mistaken for PHB granules. Subcellular localization of PHB granules within plant cells using microscopic imaging can also be problematic due to the intense fluorescence produced by the Nile Blue A polymer interaction, and researchers typically resort to transmission electron microscopy (TEM) for fine resolution of PHB subcellular localization.

Fourier-transform infrared (FTIR) microspectroscopy has proven to be a valuable and more readily available asset for identifying and localizing molecules of interest in plants [19–23]. Cell wall polysaccharides, amides, and aliphatic polyesters were measured in *Arabidopsis thaliana* petals using a FTIR microspectrometer equipped with an array mercury-cadmium-telluride detector [22]. The coupling of FTIR imaging and second derivative IR spectra revealed distinctive chemical regions such as larger quantities of aliphatic compounds and esters in the lamina, higher proportions of polysaccharides relegated to the petal stalks, and increased hemicellulosic content in the petal hinge. This instrument also enabled the differentiation of diverse mutant *Arabidopsis* plants. The use of multichannel focal plane array (FPA) detectors has not only decreased the time required to obtain high-resolution images but also allowed this imaging technique to be widely available due to the fact that measurements can be done without synchrotron sources. In addition, the technique does not require the sample to be moved on an *x*-*y* translation stage, as in IR point-mode mapping, resulting in better reproducibility. Secondary xylem in poplar and *Arabidopsis* were evaluated using a 64 × 64 FPA detector that simultaneously generated 4096 spectra [19]. The combination of this significant amount of spectral data with orthogonal projection to latent structures, a multivariate technique used to elucidate and characterize vital spectral differences using all spectral information, revealed details regarding the chemical plasticity and lignin composition at the cell level that could not have been determined using standard biochemical methods.

In the current study, a FTIR microspectrometer equipped with a 128 × 128 FPA detector was used to evaluate wild-type and PHB-containing sugarcane. This detector is capable of simultaneously generating 16,384 infrared spectra. Multivariate imaging, using principal component analysis (PCA), was used to evaluate all of the vibrational modes contained in the spectral data to hone in on the specific differences between the native and modified sugarcane.

## Results and discussion

**Fig. 2:**
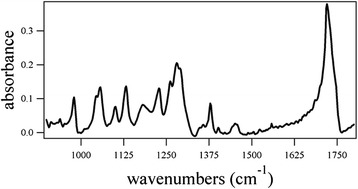
Fourier-transform infrared spectrum of poly[(*R*)-3-hydroxybutyric acid]. Vibrational modes and spectral assignments are provided in Table 1. The spectrum was collected using 256 scans and a 128 × 128 focal plane array detector

The motivation of this proof-of-concept research was to evaluate whether FTIR microspectroscopy coupled with multivariate imaging could be a valuable tool for the localization of PHB in sugarcane leaves. Poly[(*R*)-3-hydroxybutyric acid] was commercially obtained and functioned as a standard to aid in identifying vibrational modes that could provide markers to elucidate which peaks in the spectra of the sugarcane corresponded to PHB. Figure 2 shows the FTIR spectrum of PHB, and the experimental vibrational modes and spectral assignments obtained from previous PHB research are provided in Table 1.

**Table 1:** Poly[(*R*)-3-hydroxybutyric acid] vibrational modes and spectral assignments

Vibrational mode (cm^−1^)	Spectral assignment
979	C=C [31]
1045	C–CH_3_ stretching [32]
1056	C–O stretching [32]
1101	C–O–C stretching [32]
1131	CH_3_ rocking [32]
1182	C–O–C stretching [32, 33]
1228	C–O–C stretching [32, 33], CH_2_ wagging [31]
1262	C–O–C stretching + CH deformation [32, 33]
1271	C–O–C stretching (amorphous) [32]
1280	C–O–C stretching (crystalline) [32, 33]
1290	CH deformation [32]
1356	CH deformation and CH_3_ symmetric deformation [32]
1378	CH_3_ symmetric deformation [32]
1456	CH_2_ scissoring, CH_3_ asymmetric deformation [32]
1720	C=O stretch (crystalline) [32–34]
1747	C=O stretch (amorphous) [32, 34]

**Fig. 3:**
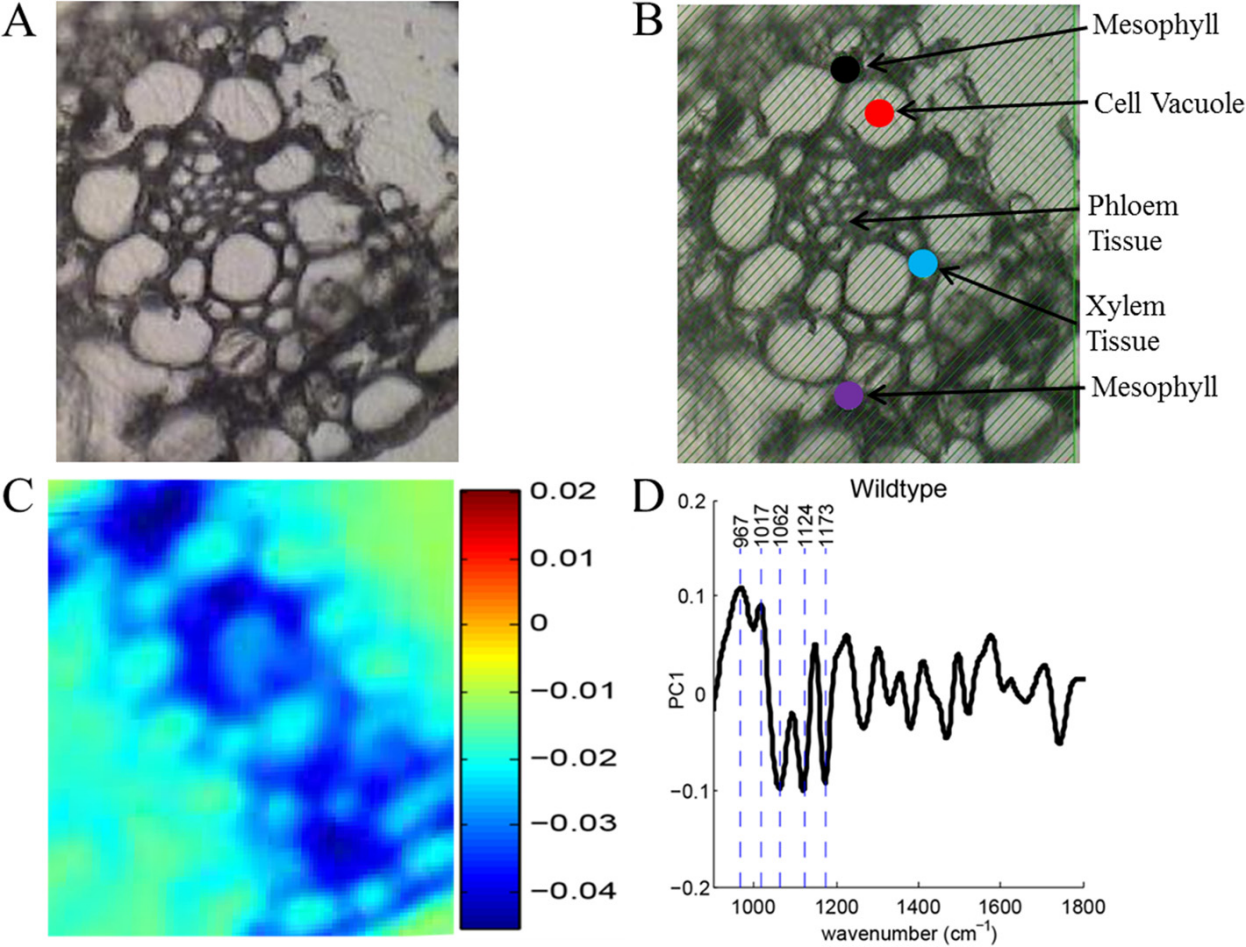
Images of wild-type sugarcane **a** without and **b** with spectral points selected. The *points* in image (**b**) correspond to the wild-type spectra in Fig. 5a. The images were collected using a 128 × 128 focal plane array detector. **c** Re-constructed image using the first principal component. **d** Loadings plot for the first principal component used in re-constructing the image in (**c**)

**Fig. 4:**
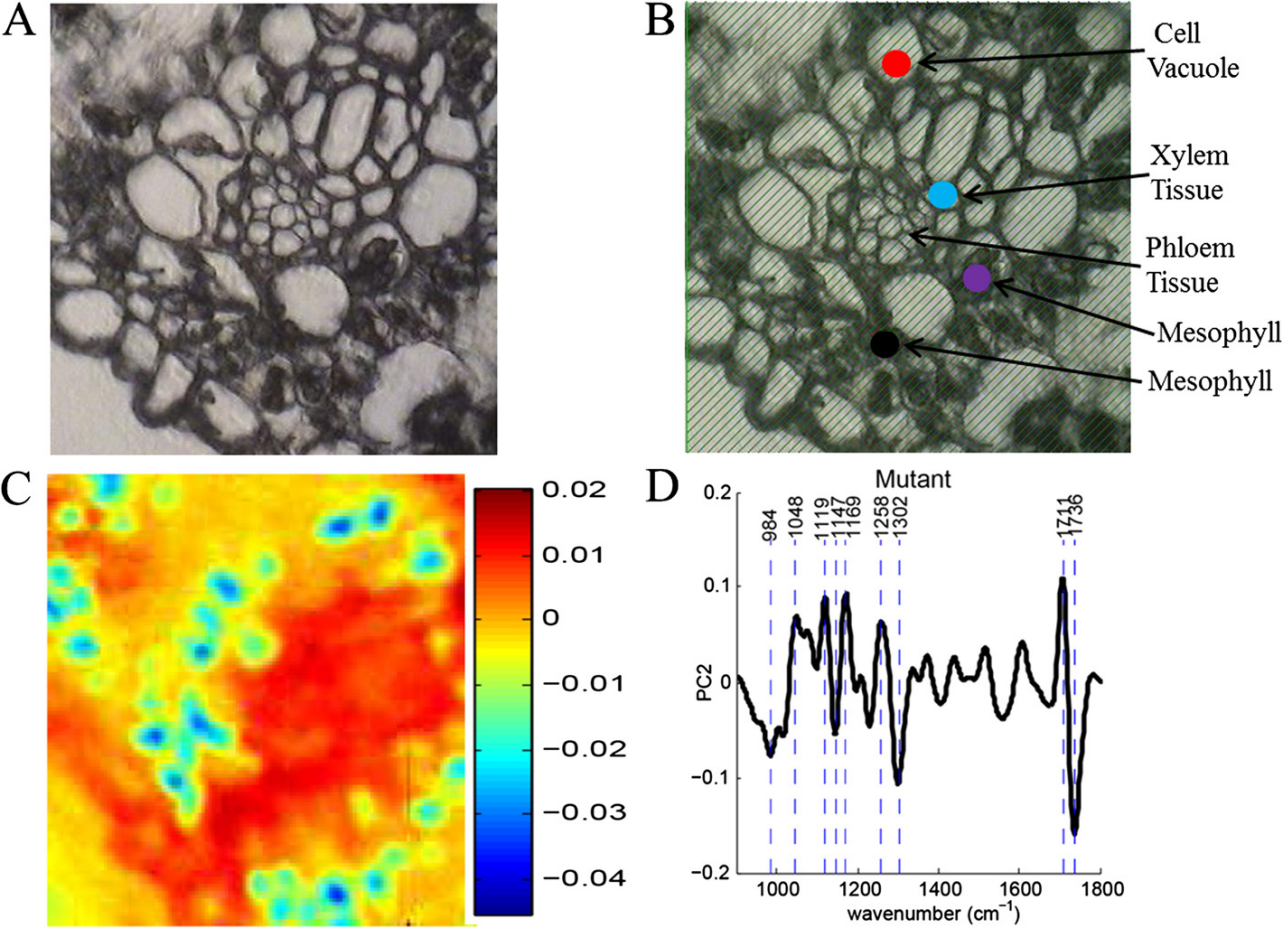
Images of polyhydroxybutyrate-containing sugarcane **a** without and **b** with spectral points selected. The *points* in image b correspond to the PHB sugarcane spectra in Fig. 5b. The images were collected using a 128 × 128 focal plane array. **c** Re-constructed image using the second principal component. **d** Loadings plot for the second principal component used in re-constructing the image in (**c**)

**Fig. 5:**
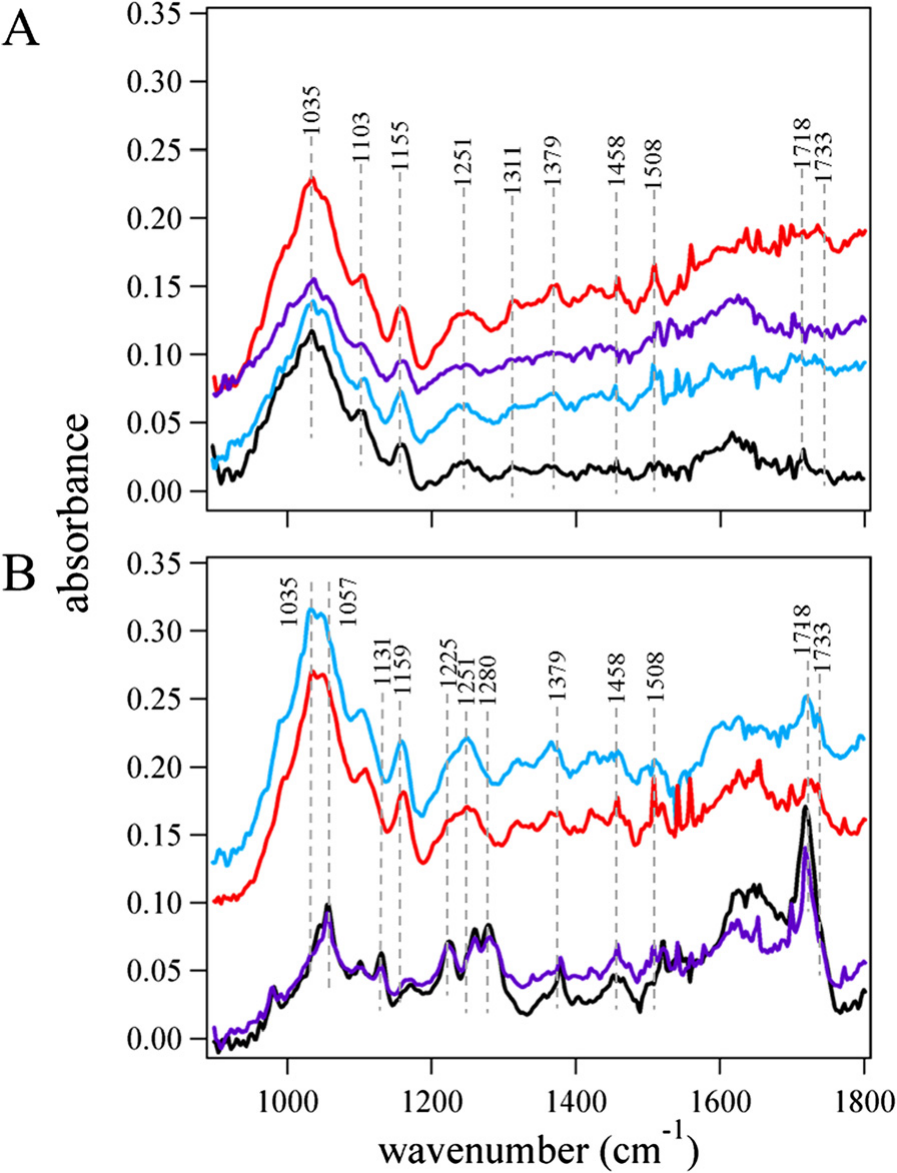
**a** Infrared spectra of wild-type sugarcane corresponding to the regions identified by the *colored circles* in Fig. 3b. **b** Infrared spectra of polyhydroxybutyrate-containing sugarcane corresponding to the regions identified by the *colored circles* in Fig. 4b. The *red spectrum* was spatially offset to facilitate a qualitative spectral analysis. All spectra were collected using 256 scans

Figures 3a and 4a provide bright-field images of wild-type and PHB sugarcane, respectively. One of the powerful features of this instrumental configuration is the ability to obtain single-point spectral data for any point in the acquired image. The images depicted by Figs. 3b and 4b illustrate the use of selecting regions to generate discrete spectra from diverse regions of the plant cross section, including xylem tissue, mesophyll, and a cell vacuole. The analysis of these images and the corresponding spectral data (Fig. 5) from the points selected exposed significant distinctions. The cross-sectional bright-field images of wild-type and PHB sugarcane in Figs. 3a and 4a revealed dark, mesophyll regions. The representative spectra from the PHB sugarcane sample (Fig. 5b) showed a C=O stretching mode near 1720 cm^−1^, which was also measured as the strongest peak in the PHB standard (Fig. 2). While this peak is not of equivalent intensity in the spectrum of wild-type sugarcane, vibrational modes corresponding to C=O stretching in lignin and acetyl groups in hemicellulose have been previously measured (Table 2). Therefore, although the intensity of this peak in the PHB sugarcane spectra is conceivably due to PHB, the analysis of other key vibrational modes is necessary. Additional analyses of the peaks in the spectra from the dark regions in the PHB sugarcane samples revealed that each peak corresponded with the expected vibrational modes identified in the PHB standard spectrum. These peaks include the following: In the PHB sugarcane leaf tissue, plausible PHB spectra were identified in mesophyll chloroplasts (Figs. 3b and 4b, black and purple circles and corresponding spectra in Fig. 5) but not in xylem tissue (Figs. 3b and 4b, blue dot and Fig. 5, blue spectrum). This concurs with previous observations based on Nile Blue A staining and TEM of PHB sugarcane leaf tissue which shows PHB localized to mesophyll chloroplasts but not xylem tissue [15, 16, 24]. Nile Blue A staining, however, is a general lipid stain and is not specific to PHB. Identification of analytes of interest can be hindered by the strong fluorescence generated, causing overexposure of the image (Fig. 1).

**Table 2:** Sugarcane cell wall vibrational modes and spectral assignments

Vibrational mode (cm^−1^)	Spectral assignment
967	Arabinoxylan [26]
991	C–O stretch [35]
1006	C–O and C–C stretch, and CH_2_ rock in cellulose [36]
1016	Pectin [27]
1026	C–O, C–O–H, C–O–C, C–C, ring stretching vibration in cellulose and hemicellulose [30, 37, 38]
1035	Aromatic C–H in-plane deformation (lignin) [19, 39];
	C–C, C–O, C–C–O, C–O–C, C–O–H stretch in cellulose and hemicellulose [19, 37, 40, 41]
1040	C–C, C–O, C–O–C, C–O–H stretching vibration in cellulose and hemicellulose [19, 37]
1053	C–O stretch, C–O, C–C ring [19, 41]
1062	Glycosidic linkage in cellulose [28]
1104	C–O–H, C–O–C, C–C, ring stretching vibration in cellulose [19, 37, 41];
	OH band in cellulose and hemicellulose [19, 38, 41]
1110	O–H band in cellulose and hemicellulose (crystalline cellulose); cellulose anti-symmetrical stretch [35]
1123	C–O, C–C stretching in starch [29]
1159	C–C, C–O, C–O–C, C–O–H stretching vibration in cellulose [19, 37, 40–42];
	Anti-symmetrical bridge oxygen stretching [35]
1173	C–O–C, C–C stretch in xylan [30]
1249	Aryl ring breathing mode; C–O stretch (lignin) [19, 35, 41];
	C–O stretch in hemicelluloses [19, 41, 42]
1315	C–H ring, CH_2_ symmetrical wagging, O–H in-plane bending [19, 35, 42]
1339	O–H in-plane bending and CH in cellulose and hemicellulose [35, 40, 42]
1374	C–H bending in cellulose [35];
	C–H, CH_2_ bending in hemicellulose [19, 41]
1419	CH_2_ scissoring at C(6) in cellulose [38];
	C–H deformation in hemicellulose [19, 41]
1458	O–H in-plane bending in cellulose [35];
	CH_3_ asymmetrical bending (lignin) [19, 39, 41]
1497	Aromatic ring vibration [43]
1509	C=C stretching vibration in aromatic ring of lignin [19, 38, 39, 41]
1541	N–H in amide II [44, 45]
1639	C=C, conjugated or aromatic carbonyl groups [46]
1647	C–O stretching vibration in lignin [41]
1716	C=O stretch in lignin [19]
1722	C=O stretch in lignin [19]
1733	C=O stretching vibration in acetyl groups of hemicellulose [38, 40–42]

A recently published manuscript discussed the ramifications of the localization of PHB in non-optimal sugarcane cell types [25]. The authors found that PHB produced in bundle sheath chloroplasts interfered with adenosine triphosphate (ATP) production and also affected the Calvin cycle. The use of FTIR microspectroscopy, juxtaposed to the less precise Nile Blue A staining, would have allowed the semi-quantitation of the amount of PHB being produced inside of the bundle sheath chloroplasts across a range of different transgenic plants that produced various levels of PHB. This quantitation would have enabled the determination of the threshold at which PHB hinders ATP in bundle sheath chloroplasts. The data generated from FTIR microspectroscopy could have been used to evaluate the efficiency of different strategies aimed at shifting PHB production from undesirable or detrimental cell types to locales better suited for this type of biomaterial production. Because the bundle sheath chloroplasts can tolerate a certain amount of PHB accumulation before ATP production is compromised [25], various plant promoters could be used, together with FTIR microspectroscopy, to identify the optimal ratio for sharing PHB production between bundle sheath and mesophyll chloroplasts and thus optimize PHB yield and biomass. This is just one example of how this technique can aid in the production of better biomaterials.

In order to characterize the differences between wild-type and PHB-containing sugarcane, unsupervised multivariate analysis was employed. The use of PCA alleviated the need to integrate specific PHB peaks that may be obscured by other cell constituents, particularly cell wall polysaccharides, by assessing all spectral information. Additionally, integrating the area under a specific peak requires spectral processing, such as baseline correction or normalization. These transformations can artificially alter the data if not carefully performed. To eliminate spectral variations such as baseline offsets, without needing to perform baseline corrections, the first derivatives were calculated for all spectra and were used in the PCA. The resulting principal component (PC) scores were then plotted according to spatial coordinates, generating images reflecting the major features contributing to specific PC loadings. The first 12 PC scores and loadings representing approximately 90 % of the variation in the samples were plotted and evaluated for the wild-type and PHB-containing sugarcane cross sections (data not shown). It must be noted that following the transformation of raw spectral data to the corresponding first derivative, there is a slight shift of the vibrational mode location along the *x*-axis. This is due to the calculation of the first derivative, as the raw peak maxima are now located at zero. The shifted vibrational modes can still be interpreted analogously to the complementary raw spectral data.

**Fig. 6:**
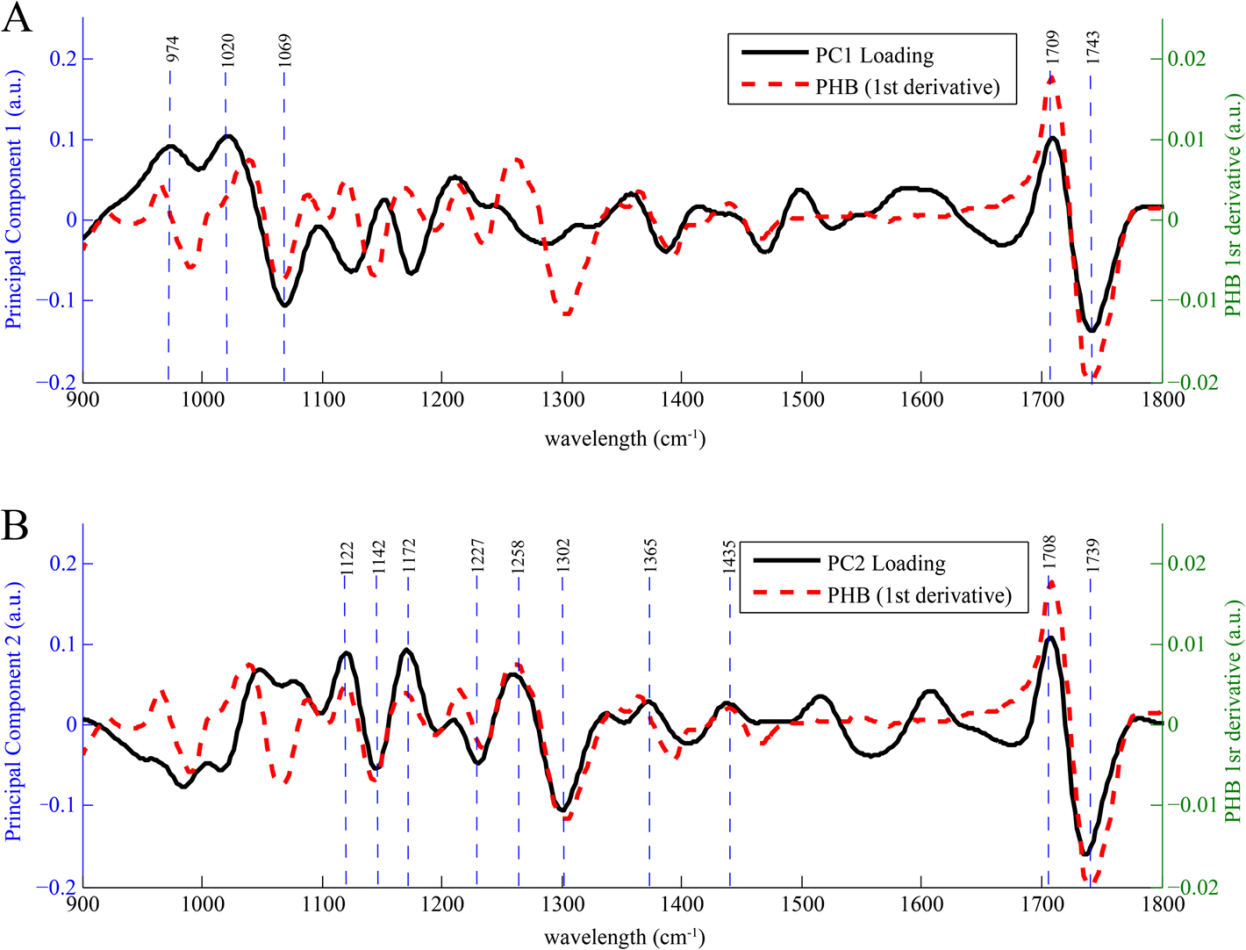
**a** First derivative spectrum of sugarcane containing PHB overlaid with the first principal component. **b** First derivative spectrum of sugarcane containing PHB overlaid with the second principal component

Figure 3c shows the heat map for the first PC scores and loadings, determined from the wild-type sugarcane first derivative spectra. Figure 3d provides the complementary loadings plot for this PC, which enabled the identification of the specific vibrational modes paramount to re-constructing the original image. Peaks near 967 cm^−1^ (arabinoxylan) [26], 1016 cm^−1^ (pectin) [27], 1062 cm^−1^ (glycoside linkage in cellulose) [28], 1123 cm^−1^ (C–O and C–C stretching in starch) [29], and 1173 cm^−1^ (xylan) [30] were identified from the first derivative IR spectra as being especially useful in producing the heat map. In comparison with the native sugarcane sample, sugarcane containing PHB, the heat maps (Fig. 4c) and loadings plot (Fig. 4d) corresponding to the second PC revealed distinctively different integral vibrational modes. The loadings plot illustrated that the PCA was converging on peaks particular to PHB. This can be further visualized by overlaying the first derivative spectrum of the PHB standard with the first and second PCs (Fig. 6a, b, respectively). Again, these vibrational modes are slightly shifted, compared to the raw spectral peaks provided in Tables 1 and 2, due to the first derivative transformation. Therefore, the first derivative PHB spectrum has been overlaid with the loadings of PC1 (Fig. 6a) and PC2 (Fig. 6b) to facilitate the analysis of the loadings. It is clear from this appraisal that the first PC, which explains 34.6 % of the spectral variance, is drawing out the 1069, 1709, and 1743 cm^−1^ PHB peaks, with other peaks at 972 and 1020 cm^−1^ indicative of cell wall constituents such as arabinoxylan and cellulose. The second PC, explaining 24.9 % of the spectral variance, however, seems to be honing in more directly to the PHB vibrational modes: 1122, 1142, 1172, 1227, 1258, 1302, 1365, 1435, 1708, and 1739 cm^−1^. Given the chemical complexity of plant samples and despite the 4 cm^−1^ spectral resolution, some of these peaks may overlap with those generated by other cell wall constituents. For example, the raw vibrational modes listed in Table 2 reveal lignin and hemicellulose peaks near 1716, 1722, and 1739 cm^−1^. The raw spectral data generated from specific plant organelles shown in Fig. 5 reveals that these C=O vibrational modes, although present, do not have considerable spectral intensities relative to the intense C=O stretch in PHB. Overlapping spectral bands necessitate the consideration of all vibrational modes being drawn out by a specific PC. The analysis of PCs beyond the first two did not prove to be useful in classifying the wild-type or PHB sugarcane samples.

## Conclusions

FTIR microspectroscopy coupled with multivariate imaging provides a powerful analytical tool for understanding plant cell ultrastructure and enables the localization of intrinsic or exogenously added molecules of interest. In this study, PHB was identified in mesophyll and bundle sheath chloroplasts. Earlier analyses of these PHB-expressing plants have relied upon staining with Nile Blue to locate the PHB. However, this stain is not specific for PHB as it is a general lipid stain. The IR method used here allows much more specific localization of PHB within the cells and tissues of the plant. For example, very specific expression in plastids is indicated by the IR method. FTIR microspectroscopy also has advantages over TEM, as the instrumentation is cheaper and less complex and does not require more experienced researchers to prepare and analyze the samples. Other components of the plant cell can also be localized using this instrumental technique, such as cellulose, hemicelluloses, and lignin, although the signal obtained from lignin vibrational modes can be rather weak when infrared excitation sources are used.

## Methods

### Sugarcane samples

The polyhydroxybutyrate-producing sugarcane line NBC37 used in this study has been previously described [16]. NBC37 and wild-type sugarcane plants of cultivar Q117 were grown to maturity in large pots containing a commercial potting mix, under glasshouse conditions at the University of Queensland, Brisbane, Australia. Fully developed whole leaf material was collected and dried in an oven at 70 °C for 48 h.

### Plant sectioning

Prior to sectioning the sugarcane, leaf samples were embedded in Tissue-Tek® O.C.T. Compound (Sakura-Finetek, Torrance, CA, USA). The samples were placed in the embedding media such that a cross section of the leaf was obtained. Additional media was spiraled around the sugarcane samples such that the sample was completely enveloped.

The plant sections were obtained using a Leica CM3050-S cryostat (Leica, Buffalo Grove, IL, USA). The temperature of the cryostat was set to −28 °C, while the sample thickness was fixed at 5 μm. The samples were affixed to standard glass microscope slides (VWR International, West Chester, Pennsylvania, USA). The O.C.T. Compound was removed from the slide by incubating the slide in a Petri dish containing deionized water, until the media was solubilized, and the sections were detached from the microscope slide. The sugarcane samples were then removed from the Petri dish using a pipette with a wide-orifice tip and placed onto optically transparent calcium fluoride windows (International Crystal Labs, Garfield, NJ, USA). The samples were put inside of a desiccator overnight to remove residual water.

### Fourier-transform infrared microspectroscopy

The FTIR imaging instrument consisted of a Bruker Hyperion 3000 infrared microscope (Bruker Corporation, Billerica, MA, USA) coupled with a Bruker Tensor 27 FTIR spectrometer (Bruker Corporation, Billerica, MA, USA). The microscope was equipped with a liquid nitrogen cooled, 128 × 128 pixel, focal plane array (FPA) detector (model# SBF161, Santa Barbara Focalplane, Goleta, CA, USA) and a 36× infrared objective. The detector has a pixel size of 40 μm, which results in a measured area of 1.1 μm per pixel. This number is the pixel resolution of the image. Using the 36× objective, the imaged area is approximately 142 μm per frame. Multiple frames were employed to provide a larger image of the sugarcane cross section. The instrumental signal was optimized prior to collecting the spectra and images by subtly re-aligning the beam path such that the maximum available signal was used. All spectral data was collected using 256 scans and a spectral resolution of 4 cm^−1^. The cross-sectional images were obtained, using transmission mode, after 14 min of analysis time. Poly[(*R*)-3-hydroxybutyric acid] (Sigma-Aldrich, St. Louis, MO, USA) was measured to identify possible vibrational modes that could be used as markers for the detection of PHB inside the sugarcane cross sections. The spectral data was imported into IGOR Pro V 6.1 (WaveMetrics Inc., Lake Oswego, OR, USA) for further analysis and plotting.

### Multivariate imaging

The first derivatives of the spectra were calculated using a Savitzky-Golay filter and 17 point smoothing. This was done to remove baseline correction issues that could occur with varying sample thickness across the plant cross sections. The data were also mean-centered. PCA was then used for data compression, and the resulting principal component (PC) scores were individually plotted according to spatial coordinates, generating an image based on each PC. All data processing was performed using custom scripts run on Matlab (Mathworks, Natick, MA, USA).

## Abbreviations

FPA: focal plane array
FTIR: Fourier-transform infrared
PC: principal component
PCA: principal component analysis
PHB: polyhydroxybutyrate
TEM: transmission electron microscopy
